# Effect of delayed diagnosis on neuroendocrine function in individuals with suprasellar germ cell tumors

**DOI:** 10.3389/fendo.2024.1408065

**Published:** 2024-06-18

**Authors:** Tao Tong, Jian Xu, Han Chen, Caiyan Mo, Dan Liang, Liyong Zhong

**Affiliations:** ^1^ Department of Endocrinology, Beijing Tiantan Hospital, Capital Medical University, Beijing, China; ^2^ Department of Geriatrics, Beijing Jishuitan Hospital, Capital Medical University, Beijing, China

**Keywords:** neuroendocrine function, delayed diagnosis, endocrinological symptoms, intracranial germ cell tumors, children and adolescents

## Abstract

**Purpose:**

The impact of delayed diagnosis on tumor-related prognosis appears to be minimal in individuals with intracranial germ cell tumors (iGCTs). However, its effect on neuroendocrine functions remains unclear. We aimed to assess the effects of delayed diagnosis on neuroendocrine function in individuals with suprasellar GCTs.

**Methods:**

We conducted a retrospective cohort study of 459 individuals with suprasellar GCTs and categorized them into two groups based on disease duration: delayed diagnosis (> 6 months) and non-delayed diagnosis (≤ 6 months). We compared endocrinological symptoms, neuroendocrine dysfunction and its grading (categorized into 0–3 grades based on severity), and recovery from neuroendocrine dysfunction in both groups.

**Results:**

Patients with delayed diagnosis exhibited higher incidences of amenorrhea, slow growth, fatigue, and polyuria/polydipsia. Neuroendocrine dysfunction, including central adrenal insufficiency (CAI), central hypothyroidism (CHT), arginine vasopressin deficiency (AVP-D), growth hormone deficiency, hypogonadism, and hyperprolactinemia, was more pronounced in the delayed diagnosis group at diagnosis, the end of treatment, and the last follow-up. Furthermore, individuals with delayed diagnosis showed higher grades of neuroendocrine dysfunction at diagnosis (OR=3.005, 95% CI 1.929–4.845, p<0.001), end of oncologic treatment (OR=4.802, 95% CI 2.878–8.004, p<0.001), and last follow-up(OR=2.335, 95% CI 1.307–4.170, p=0.005) after adjusting for confounders. Finally, less recovery, particularly in CAI, CHT, and AVP-D, was seen among the group with delayed diagnosis after treatment.

**Conclusion:**

Among individuals with suprasellar GCTs, delayed diagnosis is associated with increased, more severe, and less recovered neuroendocrine dysfunction, emphasizing the importance of early diagnosis and treatment to reduce neuroendocrine dysfunction.

## Introduction

Intracranial germ cell tumors (iGCTs) represent rare cancers with varying global incidence. Rates in Europe and the U.S. range from 0.6 to 1 per million, whereas in Japan, it is reported as 2.7 per million (1, 2). iGCTs predominantly affect the pediatric and adolescent populations, with the highest occurrence typically observed at approximately 10–12 years of age (1, 3). The WHO categorizes iGCTs as germinomas or non-germinomatous germ cell tumors (NGGCTs), including various subtypes (4). iGCTs, commonly found in the pineal and suprasellar regions, present with diverse clinical symptoms depending on the location (5–7). Pineal tumors often lead to increased intracranial pressure symptoms, such as headaches and nausea, due to obstructive hydrocephalus (8). Suprasellar region tumors typically cause endocrinological manifestations including polyuria, growth retardation, amenorrhea, delaying puberty onset, or precocious puberty (9). Visual impairment may occur if the tumor compresses the optic chiasm. “Bifocal” cases (involving both pineal and suprasellar regions simultaneously) exhibit a combination of symptoms from both regions. Additionally, a minority of patients can experience neurological symptoms, such as motor impairment, disturbance of consciousness, and epilepsy (10, 11). The treatment strategies for iGCTs include radiotherapy, chemotherapy, and surgery (12). Germinomas that are highly responsive to chemoradiotherapy have a 5-year survival rate of over 97% (13). NGGCTs can achieve a rate of 80% with combined treatment, including surgical resection of residual tumor if required (14).

Previous studies have highlighted common delayed diagnoses in patients with iGCTs, primarily associated with endocrinological symptoms (15). Suprasellar GCTs exhibit an insidious onset compounded by their complexity and exacerbating diagnostic delays (16). Currently, research related to delayed diagnosis in patients with iGCTs mainly focuses on its relationship with tumor prognosis, and the majority of studies have found that delayed diagnosis has no significant impact on patient survival or tumor recurrence rate (15, 17, 18). Extended survival increases the demand for improved quality of life, with neuroendocrine dysfunction being a key factor affecting long-term well-being (19, 20). Regrettably, existing studies have inadequately addressed neuroendocrine dysfunction, with a noticeable lack of research on how delayed diagnosis affects neuroendocrine function in patients with iGCTs. Limited studies have briefly discussed the link between delayed diagnosis and endocrinological symptoms. However, owing to the complexity of the disease and limited sample size, the conclusions are inconsistent. Hayden et al. (15) reported a higher incidence of central diabetes insipidus in 86 cases of iGCTs in patients with delayed diagnosis, whereas Jabłońska et al. (11), including 35 individuals with iGCTs, did not observe this phenomenon. Moreover, the inclusion of both suprasellar and non-suprasellar GCTs in both studies introduces a significant confounding factor, given that suprasellar GCTs are inherently associated with delayed diagnosis and neuroendocrine dysfunction (16, 21). Therefore, we explored the effect of delayed diagnosis on neuroendocrine function by reviewing a large sample of clinical data from patients with suprasellar GCTs at a single center.

## Materials and methods

### Patients

**Figure 1:**
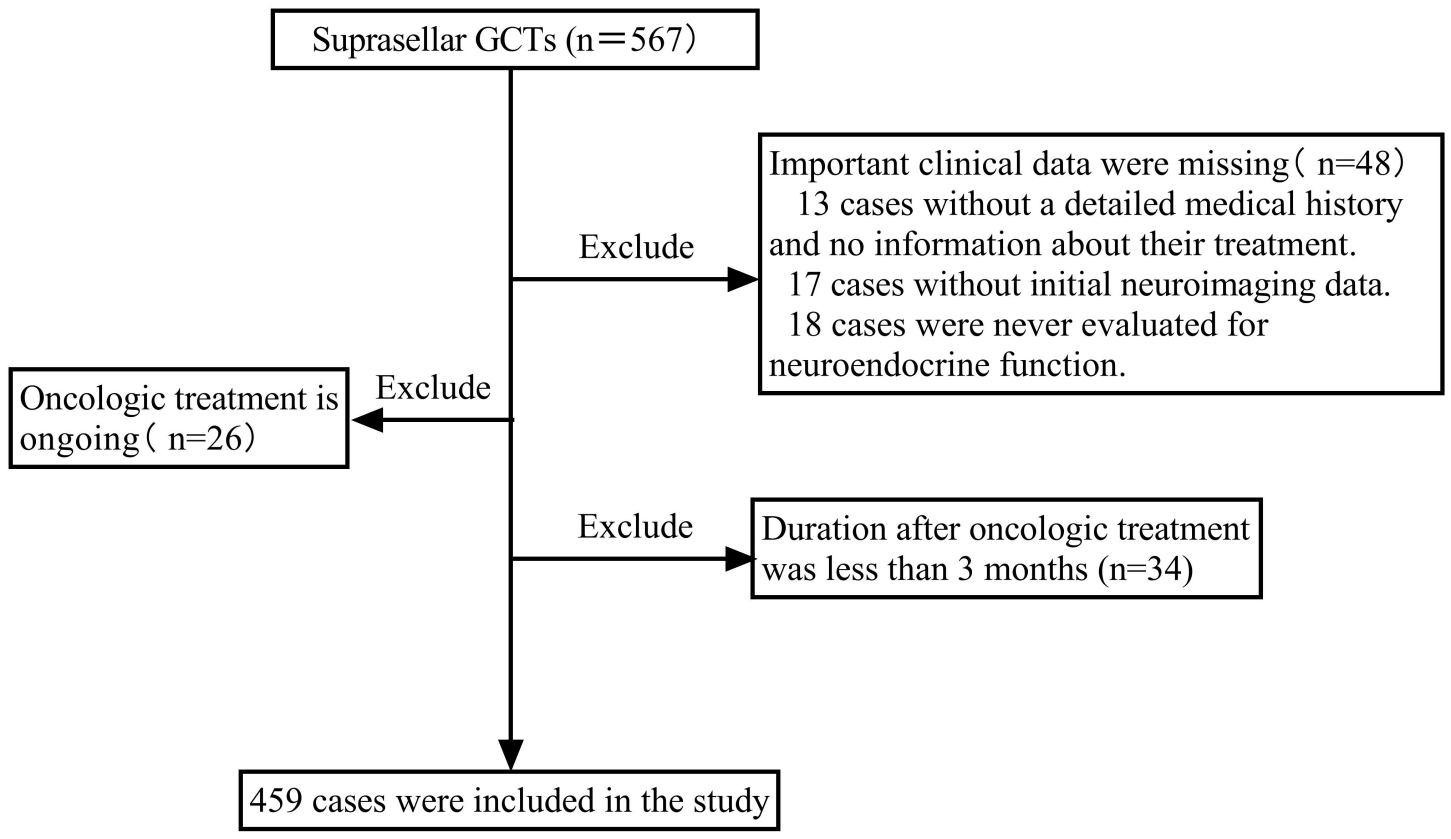
Flow chart depicting study participant inclusion. GCTs, germ cell tumors.

A retrospective cohort investigation was carried out on consecutive patients with suprasellar GCTs admitted to Beijing Tiantan Hospital, Capital Medical University, between September 2015 and August 2023, who required an endocrine follow-up period of at least 3 months. Patients who lacked important clinical data, were receiving oncologic treatment, or had completed less than 3 months of oncologic treatment were excluded. During this period, a total of 567 patients with suprasellar GCTs were identified. Among them, 48 patients were excluded due to a lack of essential clinical data. This included 13 cases with unclear medical history and treatment information, 17 cases lacking initial imaging data, and 18 cases without any neuroendocrine function assessment data. Additionally, 26 patients undergoing oncologic treatment and 34 patients receiving treatment for less than 3 months were excluded because they could not accurately assess post-treatment neuroendocrine function. Finally, 459 patients with suprasellar GCTs were enrolled, as depicted in the screening flowchart presented in **Figure 1**. In prior research on iGCTs, delayed diagnosis was considered to be the period from the first presentation to the definitive diagnosis lasting more than 6 months (11, 15, 16, 22). Therefore, this study divided all individuals into two groups based on the duration from the onset of the first symptom to definitive diagnosis: delayed diagnosis (>6 months) and non-delayed diagnosis (≤6 months). Data were collected retrospectively, including sex, age at onset, clinical symptoms (endocrinological symptoms, neurological symptoms, and symptoms associated with mass effects), tumor characteristics (maximum tumor diameter, tumor location, type, and metastasis), diagnosis, and treatment. Neuroendocrine function assessments were recorded at diagnosis, at the end of oncologic treatment, and at the last follow-up.

### Diagnosis and treatments

In this study, suprasellar GCTs were diagnosed based on histopathological findings in 189 patients (41.2%), elevation of serum/cerebrospinal beta-human chorionic gonadotropin (β-hCG) and/or alpha-fetoprotein (AFP) levels in 223 patients (48.6%), and diagnostic radiotherapy (typical clinical characteristics and imaging findings, along with a reduction of exceeding 80% in the maximum lesion diameter following low-dose local radiation therapy) in 47 patients (10.2%). Patients who had β-hCG levels above 100 IU/L or elevated AFP were deemed to be NGGCTs if no histopathological findings were present, whereas the rest of the patients were considered to be germinomas.

After diagnosis, patients underwent 2 cycles of platinum-based induction chemotherapy (ifosfamide, 1.5 g/m2, d1–3; etoposide, 70 mg/m2; d1–3; and cisplatin, 30 mg/m2; d1–3) administered every 4 weeks. Subsequently, radiotherapy was administered, followed by an additional 2 to 4 cycles of chemotherapy. Regarding the radiation field and dose in our institute for germinoma, a whole-brain/whole-ventricle radiotherapy (24.0–30.6 Gy) was followed by a boost to the tumor region for a total dose of 40 Gy. For NGGCTs, whole-brain/whole-ventricle radiotherapy (30.6 Gy) was followed by a boost to the tumor region for a total dose of 54 Gy. Craniospinal irradiation plus boost therapy was reserved for patients with metastatic disease. In clinical practice, the specific irradiation dose and field vary depending on patient age, tumor size, tumor extent, and serum levels of β-hCG and AFP. Overall, 206 individuals (44.9%) underwent surgery, of which 109 (23.7%) underwent stereotactic biopsy, 66 (14.4%) underwent surgical resection of lesions, 13 (2.9%) underwent biopsy + endoscopic third ventriculostomy (ETV) or ventriculoperitoneal shunt (VPS), and 18 (3.9%) underwent ETV or VPS. Among the 459 patients included, 428 (93.2%) received chemotherapy and all patients underwent radiotherapy. The irradiation dose and field were recorded in 291 patients, with a median total dose of 40 Gy (range:30.4 Gy-56 Gy); 187 patients (64.3%) received whole-brain/whole-ventricle radiotherapy, while 104 (35.7%) received craniospinal irradiation.

### Neuroendocrine function evaluation

Neuroendocrine functions such as the hypothalamus-pituitary-adrenal (HPA), hypothalamus-pituitary-thyroid (HPT), hypothalamus-pituitary-gonadal (HPG), growth hormone (GH)/insulin-like growth factor I (IGF-1), hypothalamus-pituitary-prolactin axis and neurohypophysis were evaluated. Thyroid hormone and thyroid-stimulating hormone were measured using Beckman chemiluminescent immunoassay (Brea, CA, USA). Serum IGF-1, GH, adrenocorticotropic hormone, cortisol, estradiol, testosterone, luteinizing hormone (LH), follicle-stimulating hormone (FSH), and prolactin were determined using chemiluminescent immunoassays (2000xpi system, SIEMENS). Cortisol serum levels below 3 µg/dL at 8:00 am or peak cortisol levels of less than 18 µg/dL during an insulin-induced hypoglycemia test were considered diagnostic of central adrenal insufficiency (CAI) (23). Central hypothyroidism (CHT) was defined as low free thyroxine and low or normal thyrotropin levels. Central hypogonadism was diagnosed in females over 13 years old and males over 14 years old when serum estradiol or testosterone levels were decreased, accompanied by low levels of LH and FSH (23). However, HPG axis function was not evaluated in patients without puberty initiation in children (males <9 years old; females <8 years old) or those in early pubertal stages (9–14 years in males; 8–13 years in females) (24). Growth hormone deficiency (GHD) was defined as a serum IGF-1 level lower than the normal reference range for age and sex, accompanied by a concomitant lack of three other pituitary hormones, or peak GH levels <5.0 μg/L in the insulin hypoglycemia stimulation test (23). After excluding stress and medication factors, prolactin serum levels exceeding the upper limit of normal reference values were considered hyperprolactinemia (HPL). Arginine vasopressin deficiency (AVP-D) was diagnosed when plasma osmolality was >295 mOsm/kg and corresponding urine osmolality was <300 mOsm/kg in fluid deprivation tests, and the symptoms of polyuria/polydipsia were alleviated after administration of vasopressin (23, 25). Precocious puberty refers to the early development of secondary sexual characteristics before the age of 8 in girls and 9 in boys (24, 26). Delayed puberty was characterized by a lack of puberty initiation until age 13 for girls and 14 for boys (27). The timing of neuroendocrine function assessment at diagnosis occurred prior to any treatment, including radiotherapy, chemotherapy, and surgery. The neuroendocrine assessment at the end of oncologic treatment was conducted within one month after completing tumor treatment. Routine follow-up assessments were conducted every 3–6 months during the initial 2 years following oncologic treatment, and thereafter, every 6–12 months. Patients were followed-up until December 2023.

### The grading of neuroendocrine dysfunction

Recognizing the significant influence of impaired neuroendocrine function on patient survival and quality of life, we implemented a four-grade classification system to assess the severity of neuroendocrine dysfunction. We also delineated the most prevalent types of pituitary dysfunction in different grades to guide the clinical practice. Grade 0 indicated normal neuroendocrine function with no impairment of the adenohypophysis or neurohypophysis function. Grade 1 indicated mild impairment in the presence of one or more components of GHD, CHG, HPL, and AVP-D. The primary type of pituitary dysfunction in this grade was AVP-D with or without one or more components of GHD, CHG, or HPL (denoted as AVP-D +/- GHD/CHG/HPL). Grade 2 signified moderate impairment in the presence of CHT, excluding CAI, with or without one or more components of GHD, CHG, HPL, or AVP-D. The most common pituitary dysfunction in grade 2 was the combination of CHT with AVP-D with or without one or more components of GHD, CHG, and HPL (indicated as CHT+AVP-D+/-GHD/CHG/HPL). Grade 3 indicated severe impairment in the presence of CAI with or without one or more components of CHT, GHD, CHG, HPL, and AVP-D. Within grade 3, panhypopituitarism (CAI+CHT+ AVP-D +GHD+CHG) was the primary type of pituitary dysfunction.

### Statistical analyses

For continuous variables that were not normally distributed, the median and interquartile range were reported, and the Mann-Whitney U test was used to perform comparisons. Categorical variables are presented as numerical values and percentages, and comparisons were performed using chi-squared or Fisher’s exact tests. To explore the independent effect of delayed diagnosis on the grade of neuroendocrine dysfunction at various stages, we conducted univariate and multivariate ordinal logistic regression analyses. In this analysis, the grade of neuroendocrine dysfunction was considered the dependent variable, while delayed/non-delayed diagnosis was treated as the independent variable. Our analyses meticulously accounted for potential confounding variables, like age of onset, sex, tumor type, tumor location, and maximum tumor diameter at diagnosis. We additionally adjusted for surgery at the end of oncologic treatment. Furthermore, the irradiation dose and those potential confounding factors mentioned above were adjusted at the last follow-up. Given that only 291 patients had detailed records of radiotherapy doses in this study, the multivariate ordinal logistic regression analyses at the last follow-up included only these 291 patients. A 2-tailed P value <0.05 was considered statistically significant. SPSS 25.0 (SPSS Inc.) was used for all statistical analyses.

## Results

### Comparison of baseline characteristics and clinical symptoms between two groups

Based on the duration from initial symptoms to definitive diagnosis, 325 patients (70.8%) were assigned to the group with delayed diagnosis, while 134 (29.2%) were in the group with non-delayed diagnosis. Of the 459 enrolled patients, 244 (53.2%) were female and 106 (23.1%) exhibited “bifocal” lesions. The median age at onset and maximum tumor diameter were 11 (8–15) years and 20 (12–32) mm, respectively. A total of 350 patients (76.3%) were diagnosed with germinoma and 109 (23.7%) were diagnosed with NGGCTs. Tumor dissemination was observed in 79 patients (17.2%). The median endocrine follow-up duration was 24 months. There were 183 patients (40.0%) with follow-up durations exceeding 36 months, 123 cases (26.8%) exceeding 48 months, 92 cases (20%) exceeding 60 months, and 61 cases (13.3%) exceeding 72 months. During the follow-up, 19 patients (4.1%) experienced a relapse, among which 2 cases died due to disease progression. Among the 17 surviving patients, 14 underwent additional radiotherapy and chemotherapy, while 3 patients received a combination of chemoradiotherapy along with surgical resection of residual lesions. Individuals with delayed diagnosis had more females, more isolated suprasellar lesions, a larger maximum tumor diameter, and a greater prevalence of germinomas than those with non-delayed diagnosis (**Table 1**).

**Table 1:** Baseline characteristics and clinical symptoms of patients with suprasellar GCTs between the delayed and non-delayed diagnosis group.

	All (N=459) n (%)	Delayed (N=325) n (%)	Non-delayed (N=134) n (%)	P value
Age at onset (year)	11 (8–15)	11 (8–14)	12 (9–16)	0.066
Female sex	244 (53.2)	192 (59.1)	52 (38.8)	<0.001
Tumor location				<0.001
Isolated suprasellar	353 (76.9)	270 (83.1)	83 (61.9)	
Bifocal	106 (23.1)	55 (16.9)	51 (38.1)	
Maximum tumor diameter (mm)	20 (12–32)	22 (15–33)	15 (9–26)	<0.001
Tumor type				0.014
Germinoma	350 (76.3)	258 (79.4)	92 (68.7)	
NGGCT	109 (23.7)	67 (20.6)	42 (31.3)	
Tumor dissemination	79 (17.2)	51 (15.7)	28 (20.9)	0.179
Endocrinological symptoms				
Polyuria or polydipsia	407 (88.7)	306 (94.2)	101 (75.4)	<0.001
Slow growth	109 (23.7)	97 (29.8)	12 (9.0)	<0.001
Amenorrhea	69 (15.0)	59 (18.2)	10 (7.5)	0.004
Delay puberty	43 (9.4)	36 (11.1)	7 (5.2)	0.050
Precocious puberty	17 (3.7)	8 (2.5)	9 (6.7)	0.028
Fatigue	99 (21.6)	79 (24.3)	20 (14.9)	0.026
Mass effects				
Visual acuity/ field changes	145 (31.6)	107 (32.9)	38 (28.4)	0.339
Headache	117 (25.5)	60 (18.5)	57 (42.5)	<0.001
Nausea/vomiting	70 (15.3)	42 (12.9)	28 (20.9)	0.031
Neurological symptoms				
Drowsiness or coma	29 (6.3)	21 (6.5)	8 (6.0)	0.844
Motor impairment	8 (1.7)	6 (1.8)	2 (1.5)	0.792
Epilepsy	6 (1.3)	4 (1.2)	2 (1.5)	0.822

NGGCT, non-germinomatous germ cell tumor.

**Figure 2:**
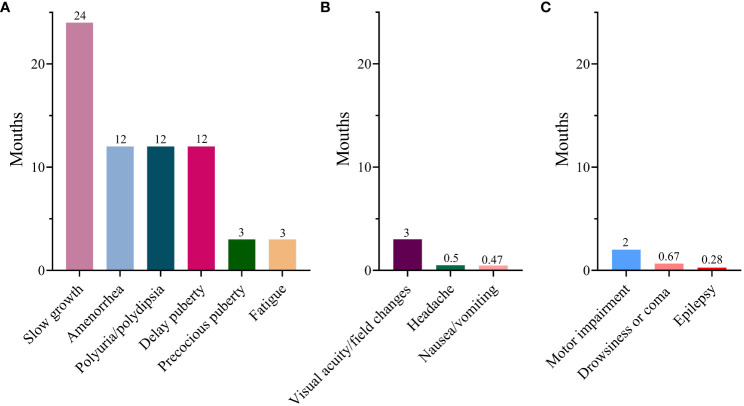
Median duration of various symptoms. **(A)** Endocrinological symptoms; **(B)** Mass effects; **(C)** Neurological symptom.

In terms of endocrinological symptoms, polyuria/polydipsia was reported in 407 cases (88.7%), slow growth in 109 (23.3%), fatigue in 99 (21.6%), amenorrhea in 69 (15%), delayed puberty in 43 (9.4%), and precocious puberty in 17 (3.7%). Regarding the clinical manifestation indicative of mass effects, visual acuity/field changes were noted in 145 cases (31.6%), headaches in 117 (25.5%), and nausea or vomiting in 70 (15.3%). However, neurological symptoms were relatively infrequent. As for the duration of symptoms, all endocrinological symptoms exhibited relatively prolonged durations. Slow growth persisted for a substantial 24-month period. Subsequently, delayed puberty, amenorrhea, and polyuria persisted for 12 months each, with fatigue being the shortest at 3 months. However, the duration of mass effects and neurological symptoms was significantly shorter than that of the endocrinological symptoms (**Figure 2**). Moreover, individuals with delayed diagnosis showed a greater frequency of endocrinological symptoms, including slow growth, polyuria/polydipsia, fatigue, and amenorrhea, when compared to those with non-delayed diagnosis. Conversely, they experienced a lower incidence of symptoms such as precocious puberty, headaches, and nausea/vomiting (**Table 1**).

### Comparison of neuroendocrine dysfunction between the two groups

A comparison of neuroendocrine dysfunction between the two groups at different stages is shown in **Table 2**. Upon diagnosis, AVP-D had the highest prevalence (96.4%), followed by CHG (83.2%), HPL (71.6%), GHD (68.8%), CAI (64.8%), and CHT (61.7%). After the end of the oncological treatment, the incidence of HPL decreased significantly to 24.5% at the last follow-up. However, the prevalence of AVP-D, CHG, GHD, CAI, and CHT remained high at the last follow-up, with rates of 93.8%, 65.3%, 71.4%, 60.8%, and 68.9%, respectively. More CAI, CHT, AVP-D, GHD, CHG, and HPL were observed in the delayed diagnosis group at diagnosis, at the end of oncologic treatment, and the last follow-up compared to those in the non-delayed diagnosis group, except for HPL at the last follow-up.

**Table 2:** Neuroendocrine dysfunction in individuals with suprasellar GCTs between the two groups at different stages.

	All n/evaluate (%)	Delayed n (%)	Non-delayed n (%)	P value
At diagnosis				
AVP-D	428/444 (96.4))	306 (97.8)	122 (93.1)	0.017
CAI	285/440 (64.8)	231 (74.3)	54 (41.9)	<0.001
CHT	272/441 (61.7)	212 (68.2)	60 (46.2)	<0.001
CHG	203/244 (83.2)	154 (88.5)	49 (70.0)	<0.001
GHD	275/400 (68.8)	217 (77.5)	58 (48.3)	<0.001
HPL	315/440 (71.6)	233 (75.2)	82 (63.1)	0.010
End of oncologic treatment				
AVP-D	373/393 (94.9)	262 (98.9)	111 (86.7)	<0.001
CAI	238/392 (60.7)	195 (73.6)	43 (33.9)	<0.001
CHT	247/393 (62.8)	194 (73.2)	53 (41.4)	<0.001
CHG	179/251 (71.3)	137 (79.7)	42 (53.2)	0.002
GHD	249/365 (68.2)	190 (76.9)	59 (50.0)	<0.001
HPL	154/393 (39.2)	118 (44.5)	36 (28.1)	0.002
Last follow up				
AVP-D	408/435 (93.8)	294 (96.4)	114 (87.7)	0.001
CAI	264/434 (60.8)	211 (69.4)	53 (40.8)	<0.001
CHT	299/434 (68.9)	235 (77.3)	64 (49.2)	<0.001
CHG	226/346 (65.3)	177 (72.2)	49 (48.5)	<0.001
GHD	299/419 (71.4)	229 (77.4)	70 (56.9)	<0.001
HPL	104/425 (24.5)	79 (26.6)	25 (19.5)	0.120

AVP-D, arginine vasopressin deficiency; CHT, central hypothyroidism; CAI, central adrenal insufficiency; GHD, growth hormone deficiency; CHG, central hypogonadism; HPL, hyperprolactinemia.

Moreover, considering the large variation in the follow-up period of patients and the time-dependent effects of radiotherapy on neuroendocrine function, we further compared the neuroendocrine function between the two groups at different follow-up periods. The results revealed that regardless of whether it was at the 24-month, 48-month, or 72-month follow-up, patients with delayed diagnosis still exhibited a higher incidence of CAI, CHT, AVP-D, GHD, and CHG than those with non-delayed diagnosis (**Table 3**).

**Table 3:** Neuroendocrine dysfunction in individuals with suprasellar GCTs between the two groups at different follow-up periods.

	At 24-month follow-up (n=239)			At 48-month follow-up (n=123)			At 72-month follow-up (n=61)		
	Delayed(n=168) n/evaluate(%)	Non-delayed(n=71) n/evaluate(%)	P value	Delayed(n=93) n/evaluate(%)	Non-delayed(n=30) n/evaluate(%)	P value	Delayed(n=43) n/evaluate(%)	Non-delayed(n=18) n/evaluate(%)	P value
AVP-D	155/162(95.7)	57/69(82.6)	0.001	83/88(94.3)	22/28(78.6)	0.013	42/43(97.7)	13/16(81.3)	0.026
CAI	102/161(63.4)	31/68(45.6)	0.013	56/88(63.6)	8/28(28.6)	0.001	26/43(60.5)	4/16(25)	0.015
CHT	115/161(71.4)	37/69(53.6)	0.009	65/88(79.3)	11/28(39.3)	0.001	29/43(67.4)	5/16(31.3)	0.012
CHG	104/140(74.3)	25/49(51.0)	0.003	61/83(73.5)	7/21(33.3)	0.001	33/42(78.6)	5/15(33.3)	0.001
GHD	123/153(80.4)	42/64(65.6)	0.020	65/82(79.3)	13/27(48.1)	0.002	28/40(70.0)	5/15(33.3)	0.013
HPL	48/157(30.6)	12/69(17.4)	0.039	23/86(26.7)	4/28(14.3)	0.178	14/41(34.1)	2/16(12.5)	0.102

AVP-D, arginine vasopressin deficiency; CHT, central hypothyroidism; CAI, central adrenal insufficiency; GHD, growth hormone deficiency; CHG, central hypogonadism; HPL, hyperprolactinemia.

### Comparison of the grades of neuroendocrine dysfunction between the two groups

A comparison of grades of neuroendocrine dysfunction and their corresponding primary types of pituitary dysfunction in the two groups at various stages is presented in **Table 4**. At diagnosis, the most common grade of neuroendocrine dysfunction was grade 3 (285 cases, 64.4%). This was followed by grades 1, 2, and 0 in the order of 123 (27.7%), 32 (7.2%), and 4 (0.9%) cases, respectively. After oncologic treatment and during the last follow-up, the prevalence of grade 3 slightly decreased, yet it remained the predominant category. Except for the lack of statistical significance in the difference in grade 0 at diagnosis or grade 2 at the last follow-up between the two groups, individuals with delayed diagnosis were more likely to have grade 3 neuroendocrine dysfunction and less likely to have grade 0–2 neuroendocrine dysfunction when compared to those with a non-delayed diagnosis at diagnosis, end of oncologic treatment, and last follow-up. Among the primary types of pituitary dysfunction among grades 1–3, aside from the absence of statistically significant disparities in CHT+ AVP-D +/- GHD/CHG/HPL between the two groups at the last follow-up, individuals with delayed diagnoses had more panhypopituitarism and a lower prevalence of AVP-D +/- GHD/CHG/HPL and CHT+ AVP-D +/- GHD/CHG/HPL, whether at the diagnosis, end of oncologic treatment, or last follow-up.

**Table 4:** The grades of neuroendocrine dysfunction and the corresponding most common types of pituitary dysfunction between the two groups at different stages.

	All n (%)	Delayed n (%)	Non-delayed n (%)	P value
**At diagnosis**	444	313	131	
**Grade 0**	4 (0.9)	2 (0.6)	2 (1.5)	0.367
**Grade 1**	123 (27.7)	63 (20.1)	60 (45.8)	<0.001
AVP-D +/- GHD/ CHG/HPL	118 (26.6)	61 (19.6)	57 (43.5)	<0.001
**Grade 2**	32 (7.2)	17 (5.4)	15 (11.5)	0.025
CHT+AVP-D+/- GHD/CHG/HPL	32 (7.2)	17 (5.4)	15 (11.5)	0.025
**Grade 3**	285 (64.4)	231 (73.8)	54 (41.2)	<0.001
CAI+CHT+ AVP- D+GHD+CHG	108/222^a^ (48.6)	94/160 (58.8)	14/62 (22.6)	<0.001
**End of oncologic treatment**	393	265	128	
**Grade 0**	9 (2.3)	0 (0.0)	9 (7.0)	<0.001
**Grade 1**	115 (29.3)	57 (21.5)	58 (45.3)	<0.001
AVP-D+/- GHD/ CHG/HPL	111 (28.2)	55 (20.8)	56 (43.8)	<0.001
**Grade 2**	31 (7.9)	13 (4.9)	18 (14.1)	0.002
CHT+AVP-D+/- GHD/CHG/HPL	26 (6.6)	12 (4.5)	14 (10.9)	0.017
**Grade 3**	238 (60.6)	195 (73.3)	43 (33.6)	<0.001
CAI+CHT+ AVP- D+GHD+CHG	121/237^a^ (51.1)	103/164 (62.8)	18/73 (24.7)	<0.001
**Last follow up**	434	304	130	
**Grade 0**	15 (3.5)	6 (2.0)	9 (6.9)	0.010
**Grade 1**	104 (24.0)	52 (17.1)	52 (40.0)	<0.001
AVP-D+/- GHD/ CHG/HPL	100 (23.0)	51 (16.8)	49 (37.7)	<0.001
**Grade 2**	51 (11.8)	35 (11.5)	16 (12.3)	0.814
CHT+AVP-D+/- GHD/CHG/HPL	45 (10.4)	32 (10.5)	13 (10.0)	0.869
**Grade 3**	264 (60.8)	211 (69.4)	53 (40.8)	<0.001
CAI+CHT+ AVP- D+GHD+CHG	157/333^a^ (47.1)	132/238 (55.5)	25/95 (26.3)	<0.001

AVP-D, arginine vasopressin deficiency; CHT, central hypothyroidism; CAI, central adrenal insufficiency; GHD, growth hormone deficiency; CHG, central hypogonadism; HPL, hyperprolactinemia; ^a^represents the number of patients evaluated for CAI, CHT, AVP-D, GHD and CHG.

### Independent effect of delayed diagnosis on grades of neuroendocrine dysfunction

To investigate the independent effect of delayed diagnosis on the grades of neuroendocrine dysfunction, we conducted an ordered logistic regression analysis. Univariate analysis revealed that individuals with delayed diagnosis exhibited higher grades of neuroendocrine dysfunction at diagnosis, end of oncologic treatment, and last follow-up. Following adjustments for other potential confounding factors, this conclusion remained evident, both at the time of diagnosis (OR=3.005, 95% CI 1.929–4.845, p<0.001), at the end of oncologic treatment (OR=4.802, 95% CI 2.878–8.004, p<0.001), and during the last follow-up (OR=2.335, 95% CI 1.307–4.170, p=0.005) (**Table 5**).

**Table 5:** Ordinal logistic regression analysis for the associations between delayed diagnosis and grade of neuroendocrine dysfunction in patients with suprasellar GCTs at different stages.

	At diagnosis		End of oncologic treatment		Last-follow up	
	OR (95 CI)	P value	OR (95 CI)	P value	OR (95 CI)	P value
Univariate analysis						
Delayed vs Non-delayed	3.739(2.472, 5.652)	<0.001	5.053(3.277, 7.791)	<0.001	3.479(2.323, 5.212)	<0.001
Non-delayed	Ref		Ref		Ref	
Adjusting other factors						
Delayed	3.055(1.929, 4.845)	<0.001^a^	4.802(2.878, 8.004)	<0.001^b^	2.335(1.307, 4.170)	0.005^c^
Non-delayed	Ref		Ref		Ref	

GCTs, germ cell tumor; OR, odds ratio; ^a^adjusted for age, sex, tumor location, tumor type, maximum tumor diameter; ^b^adjusted for age, sex, tumor location, tumor type, maximum tumor diameter, surgery; ^c^adjusted for age, sex, tumor location, tumor type, maximum tumor diameter, surgery, and radiation dose.

### Comparison of the recovery of neuroendocrine dysfunction between the two groups

**Figure 3:**
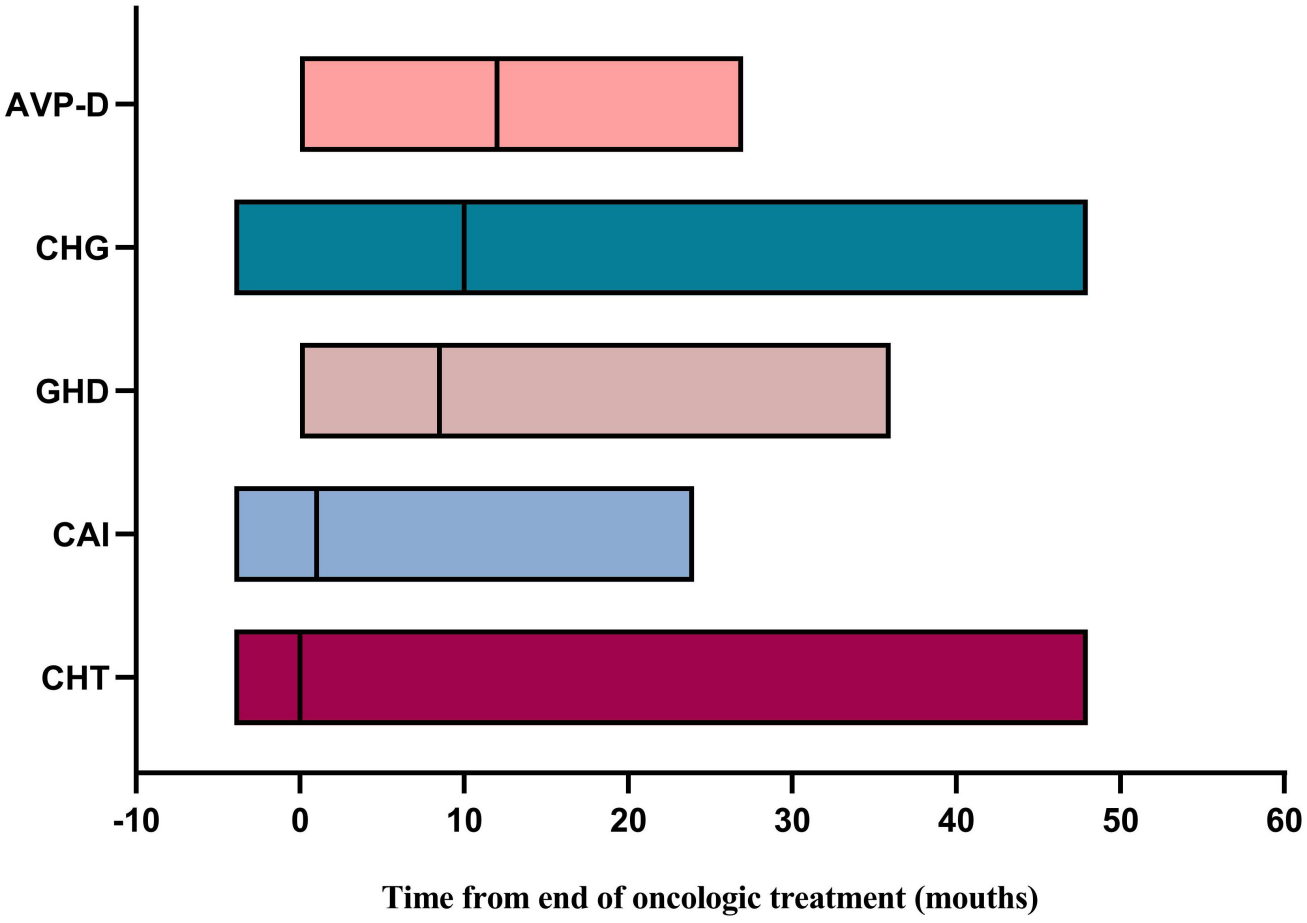
Time for recovery of neuroendocrine dysfunction from the end of oncologic treatment. AVP-D, arginine vasopressin deficiency; CHG, central hypogonadism; GHD, growth hormone deficiency; CAI, central adrenal insufficiency; CHT, central hypothyroidism.

We further explored the recovery of the impaired neuroendocrine function after treatment. The results showed that, compared to the time of diagnosis, a certain proportion of patients recovered from neuroendocrine dysfunction during follow-up. Notably, HPL exhibited the highest recovery rate of 67.3%, followed by CHG at 22.6%, CAI at 18.3%, CHT at 10.0%, and GHD at 6.1%. AVP-D displayed the lowest recovery rate of 3.7% (**Table 6**). Taking the end of oncologic treatment as the starting point, the recovery times for AVP-D, CHG, and GHD were relatively long, with a median of 12 months (range: 0–27 months), 10 months (range: -4 to 48 months), and 9 months (range: 0–36 months), respectively. In contrast, CAI and CHT had relatively short recovery times, with a median of 1 month (range: -4 to 24 months) and 0 months (range: -4 to 48 months), respectively (**Figure 3**). Individuals with a delayed diagnosis experienced significantly lower recovery rates for AVP-D, CAI, and CHT than those with a non-delayed diagnosis. Although there were also lower recovery percentages in CHG, GHD, and HPL, these differences did not reach statistical significance (**Table 6**).

**Table 6:** Recovery of neuroendocrine dysfunction between the delayed and non-delayed diagnosis groups in patients with suprasellar GCTs.

	All n/evaluate (%)	Delayed n (%)	Non-delayed n (%)	P value
Recovery of AVP-D	15/410 (3.7)	7 (2.4)	8 (6.7)	0.037
Recovery of CAI	50/273 (18.3)	35 (16.0)	15 (27.8)	0.045
Recovery of CHT	26/260 (10.0)	15 (7.5)	11 (18.3)	0.014
Recovery of CHG	42/186 (22.6)	29 (20.3)	13 (30.2)	0.171
Recovery of GHD	16/262 (6.1)	11 (5.3)	5 (8.9)	0.320
Recovery of HPL	198/294 (67.3)	141 (65.3)	57 (73.1)	0.208

AVP-D, arginine vasopressin deficiency; CHT, central hypothyroidism; CAI, central adrenal insufficiency; GHD, growth hormone deficiency; CHG, central hypogonadism; HPL, hyperprolactinemia.

During the period from diagnosis to the end of oncologic treatment, 64 patients (13.9%) developed new-onset neuroendocrine dysfunction. The incidence of newly developed AVP-D, CAI, CHT, CHG, and GHD was 4/11 (36.3%), 24/138 (17.4%), 32/154 (20.8%), 2/35 (5.7%), and 18/109 (16.5%), respectively. A higher proportion of surgical resection or biopsy was observed in newly developed cases than in those without newly developed dysfunction (53.1% vs 39.0%, p=0.033). During the follow-up period from oncologic treatment completion to the last follow-up, 55 patients (12.0%) experienced new-onset neuroendocrine dysfunction. Among them, at the follow-up time points of 24, 36, 48, 60, 72, and 84 months, there were 12 cases (2.6%), 31 cases (6.8%), 38 cases (8.3%), 43 cases (9.4%), 48 cases (10.5%), and 55 cases (12.0%), respectively. The incidences of newly developed AVP-D, CAI, CHT, CHG, and GHD were 6/27 (22.2%), 23/151 (15.2%), 24/142 (16.9%), 6/60 (10%), and 20/113 (17.7%), respectively. The median radiation dose was higher in the newly developed dysfunction group than in the non-newly developed dysfunction group (44.6Gy vs 40.0 Gy, p = 0.037).

## Discussion

To our knowledge, the present study is the largest sample of suprasellar GCTs in a single center and is the first to explore the effect of delayed diagnosis on neuroendocrine function. The results showed that 70.8% of patients encountered delayed diagnosis, and these patients exhibited more endocrinological symptoms that persisted for a prolonged duration. At diagnosis, end of oncologic treatment, or final follow-up, patients with a delayed diagnosis consistently demonstrated a higher proportion and greater severity of neuroendocrine dysfunction. Simultaneously, these patients showed a lower recovery rate of neuroendocrine function during the follow-up.

iGCTs are rare malignant tumors characterized by high clinical heterogeneity. Research on the neuroendocrine function of iGCTs is still limited, and the available studies have small sample sizes. The present study, conducted with a large sample size, demonstrated that the delayed diagnosis groups had more CAI, CHT, AVP-D, GHD, and CHG compared to patients with non-delayed diagnosis. However, one study with a small sample size did not find similar results. Chang et al. (22) discovered that individuals with delayed diagnosis exhibited more CHT at the time of diagnosis, but not AVP-D, CAI, GHD, or CHG, in a study of 49 germinoma cases. Possible reasons for this are the small number of participants and the inclusion of individuals with both suprasellar and non-suprasellar lesions, which are inherently less likely to cause neuroendocrine dysfunction. Once neuroendocrine dysfunction is identified, especially with CAI, prompt administration of glucocorticoid replacement therapy is crucial to prevent the onset of pituitary crisis or even life-threatening conditions following stress. Notably, our study revealed that the highest proportion of grade 3 neuroendocrine dysfunction was observed, with panhypopituitarism being predominant. In addition to appropriate glucocorticoid replacement therapy in patients with grade 3 neuroendocrine dysfunction, thyroxine and desmopressin replacement therapy is also indispensable when it is identified. It not only relieves patients’ clinical symptoms but also promotes the treatment of tumors during the peri-tumor treatment period. Conversely, GHD and CHG had a minimal impact on survival or quality of life during this period, suggesting that corresponding hormone replacement therapy might be unnecessary during the peri-tumor treatment period. However, these children and adolescents may face growth and pubertal development challenges associated with GHD and CHG during long-term management after the end of oncologic treatment (28). Hence, a comprehensive and proactive evaluation of neuroendocrine function, combined with accurate grading, is imperative in patients with suprasellar GCTs. As chemoradiotherapy is administered, and if necessary, combined with surgical interventions, the tumor can be effectively eliminated, leading to low recurrence rates and improved survival outcomes. However, the legacy of the neuroendocrine dysfunction persists (29–31). This study observed the persistence of varying degrees of pituitary dysfunction at the end of oncologic treatment and during the final follow-up. Furthermore, patients with delayed diagnoses exhibited a higher proportion and greater severity of neuroendocrine dysfunction throughout the disease period, even after adjusting for potential influencing factors. These findings suggest that long-term hormone replacement therapy and regular follow-up are necessary.

Prior research has established that individuals with iGCTs undergoing surgery as well as high-dose radiotherapy may develop new-onset pituitary impairment (30, 32). Fortunately, however, a subset of patients recover from pituitary dysfunction after tumor elimination (21, 33). Xiang et al. (21) reported that, after a median follow-up of 19 months, the recovery rates of CAI, CHT, AVP-D, CHG, and HPL in patients with iGCTs were 2/30 (6.6%), 7/38 (18.3%), 11/45 (24.4%), 7/33 (21.1%), and 9/23 (39.1%), respectively. Similarly, Zhang et al. (34) reported that up to 28/75 (37.3%) patients with suprasellar GCTs experienced recovery or initiation of HPG axis function, and less recovery in patients with a delayed diagnosis of ≥1.7 years. Our study also found that the recovery rates for HPL, CHG, CAI, CHT, GHD, and AVP-D at the last follow-up were 67.3%, 22.6%, 18.3%, 10.0%, 6.1%, and 3.7%, respectively. We also observed a lower recovery of CHT, CAI, and AVP-D in the delayed diagnosis group. Unlike the findings of Zhang et al. (34),our study did not observe significantly lower recovery from CHG in the delayed diagnosis group. This disparity could be explained by the fact that the definition of a delayed diagnosis was considered to be a disease duration of more than half a year in the present study, whereas it was described as 1.7 years in the study by Zhang et al. Both instances underscore that early diagnosis and treatment are advantageous in restoring neuroendocrine disturbances.

The delayed diagnosis group experienced less recovery of CAI, CHT, and AVP-D, but not of CHG, GHD, and HPL. The duration of recovery varied for each pituitary hormone deficiency condition. The reasons for these differences remain unclear; however, the diverse durations of pituitary hormone deficiency at diagnosis could be a factor. Previous small-sample surveys indicated that AVP-D in patients with iGCTs typically emerged relatively early and was difficult to recover from after treatment, persisting over time (17, 35, 36). This study also revealed that symptoms related to posterior pituitary dysfunction, such as polydipsia and polyuria, appeared early. Among these pituitary dysfunctions, AVP-D was the most prevalent, exhibiting the lowest recovery rate and the longest recovery period. These findings collectively suggest that the neurohypophysis is susceptible to damage, and recovery after tumor elimination is challenging. However, early diagnosis and treatment still hold significance for recovery. Slow growth associated with GH/IGF-1 axis impairment had the longest duration of symptoms, followed by amenorrhea and delayed puberty associated with HPG axis impairment. This finding implied that GHD and CHG persisted for an extended period until a definitive diagnosis was made. Consequently, even with early diagnosis and treatment, the likelihood of recovery from GHD and CHG diminishes, and the recovery process is relatively prolonged. Fatigue symptoms, potentially linked to HPA and HPT axis dysfunction, had the shortest duration among the endocrinological symptoms. Recovery time for CAI and CHT after treatment was relatively fast, with most patients recovering by the end of oncological treatment. Non-delayed diagnosed individuals tended to have a better chance of recovering from neuroendocrine dysfunction. These observations suggest that HPA- and HPT-axis dysfunction occurs relatively late, and early diagnosis can improve the likelihood of recovery. HPL is caused by tumor invasion into the hypothalamus or pituitary stalk, which reduces dopamine synthesis or impaired transport. Following tumor elimination through chemoradiotherapy, dopamine delivery to the pituitary gland is enhanced, thereby reducing prolactin release. Consequently, both the delayed and non-delayed diagnosis groups exhibited a high proportion of prolactin reduction after tumor resolution.

This study had several limitations. The data relied on the completeness and accuracy of medical records and admission forms. Interpretation of results, including symptom duration, depended on patient reports and the expertise of medical professionals. Due to the retrospective nature of the study, a comprehensive assessment of hypothalamic function impairment was not possible. Future research should address this limitation by including pertinent data. Moreover, the endocrine follow-up period was relatively short (only 2 years), whereas hypopituitarism typically emerges 3–5 years after radiotherapy. Extended follow-up is essential to determine whether more patients develop pituitary dysfunction.

## Conclusion

There was a significant delay in diagnosis, up to 70.8%, in individuals with suprasellar GCTs. Those with a delayed diagnosis had more endocrinological symptoms and consistently experienced a higher proportion and more severe neuroendocrine dysfunction throughout the course of the disease. Furthermore, a lower recovery of neuroendocrine function after tumor removal, especially in AVP-D, CAI, and CHT, was observed in patients with a delayed diagnosis. Therefore, early diagnosis and treatment may contribute to minimizing neuroendocrine function damage and promote better recovery of neuroendocrine function after treatment.

## Data availability statement

The raw data supporting the conclusions of this article will be made available by the authors, without undue reservation.

## Ethics statement

The studies involving humans were approved by IRB of Beijing Tiantan Hospital, Capital Medical University. The studies were conducted in accordance with the local legislation and institutional requirements. Written informed consent for participation in this study was provided by the participants’ legal guardians/next of kin. Written informed consent was obtained from the individual(s), and minor(s)’ legal guardian/next of kin, for the publication of any potentially identifiable images or data included in this article.

## Author contributions

TT: Methodology, Writing – review & editing, Data curation, Investigation, Writing – original draft. JX: Investigation, Methodology, Writing – review & editing, Conceptualization, Software. HC: Investigation, Methodology, Software, Data curation, Formal analysis, Writing – original draft. CM: Data curation, Formal analysis, Investigation, Methodology, Software, Writing – original draft. DL: Data curation, Investigation, Methodology, Software, Writing – original draft. LZ: Methodology, Conceptualization, Project administration, Supervision, Writing – review & editing.
